# Computed Tomographic Findings of Injuries After Mechanical and Manual Resuscitation: A Retrospective Study

**DOI:** 10.7759/cureus.15131

**Published:** 2021-05-20

**Authors:** Mustafa Emin Canakci, Kubra Parpucu Bagceci, Nurdan Acar, Engin Ozakin, Filiz Baloglu Kaya, Caglar Kuas, Murat Çetin, Betül Tiryaki Baştuğ, Muhammed Evvah Karakılıç

**Affiliations:** 1 Emergency Medicine, Eskisehir Osmangazi University, Eskisehir, TUR; 2 Emergency Medicine, Yunus Emre State Hospital, Eskisehir, TUR; 3 Emergency Medicine, Ankara Yenimahalle Research and Training Hospital, Eskisehir, TUR; 4 Emergency Medicine, Izmir Tinaztepe University, Izmir, TUR; 5 Radiology, Eskisehir Osmangazi University, Eskisehir, TUR

**Keywords:** cardiopulmonary resuscitation, mechanical resuscitation, emergency department, computerized tomography, in-hospital cardiac arrest

## Abstract

Introduction

Cardiopulmonary resuscitation (CPR)-related injuries are complications of chest compressions during CPR. This study aimed to investigate the differences and complications between mechanical and manual CPR techniques by using computed tomography (CT).

Methods

Patients in whom return of spontaneous circulation was achieved after CPR and thorax CT imaging were performed for diagnostic purposes were included in the study.

Results

A total of 178 non-traumatic cardiac arrest patients were successfully resuscitated and had CT scans in the emergency department. The complications of CPR are sternum fracture, rib fracture, pleural effusion/hemothorax, and pneumothorax. There were no statistically significant differences in terms of age, first complaint, cardiac arrest rhythm, CPR duration, and complications between mechanical and manual CPR. The number of exitus in the emergency department was similar (p=0.638). The discharge from hospital rate was higher in the mechanical CPR group but there was no statistically significant difference (p=0.196). The duration of CPR was associated with the number of rib fractures and lung contusion, but it did not affect other CPR-related chest injuries.

Conclusion

There was no significant difference observed in terms of increased complications in patients who received mechanical compression as compared with those who received manual compression. According to our results, mechanical compression does not cause serious complications, and the discharge from hospital rate was higher than for manual CPR; therefore, its use should be encouraged.

## Introduction

Cardiopulmonary resuscitation (CPR) is a life support procedure comprising chest compressions and artificial ventilation to maintain circulation and oxygenation during a cardiac arrest. It was described by Kouwenhoven and Jude and introduced in clinical practice in the 1960s [1]. This simple procedure can be performed by anyone, often resulting in a significant increase in the survival rates of patients with sudden cardiac arrest [2]. With the revisions in the European Resuscitation Society and American Heart Association guidelines with time, the appropriate distance for an effective CPR depth has been determined to be 5 cm and the rate to be 100-120 beats/min for average adults [3-4]. Changes in CPR depth and rate determine the frequency and severity of injury resulting from CPR. Rib and sternum fractures are the most commonly reported complications of CPR. Other notable complications include liver and spleen injury, pneumothorax, hemothorax, and cardiac laceration [5-7]. The severity of these complications is also among the important factors that determine the survival rate and outcome of these patients.

CPR can be performed by using two methods: manual and mechanical. Manual chest compression depends on several factors such as the practitioner’s environment and mental and physical strength; its effectiveness may vary depending on the individual [8]. Conversely, exhaustion of the rescuers because of prolonged CPR leads to a decrease in the effectiveness of CPR [9]. Mechanical compression devices have been invented to solve the problems that may arise from manual chest compression, standardize the CPR process, and increase CPR effectiveness [10]. These devices have different operating principles; devices that operate with the active compression-decompression principle are used most frequently. Mechanical compression devices minimize pauses during transport, allowing rescuers to focus on advanced life support [11]. In addition, they ensure the effectiveness and continuity of CPR by adjusting and standardizing parameters, such as frequency, depth, and rhythm, thus aiming to increase the effectiveness of CPR and the survival rate of patients [12]. However, mechanical CPR devices can also cause serious injuries depending on the mechanical force applied. Some studies have reported that these injuries have similar rates while using the manual and mechanical CPR methods, and there is no significant difference between the methods regarding the improvement of survival or neurological outcomes. Hence, the routine use of mechanical devices has not been recommended yet [13].

Post-CPR complications bring serious legal and medical responsibilities for physicians. Most studies examining complications after CPR are based on autopsy results and do not include the CPR method used [13-15]. This study aimed to evaluate the complications that can be detected by computed tomography (CT), survival and neurological outcomes, as well as the differences between the two methods performed in patients who experienced an in-hospital witnessed cardiac arrest.

## Materials and methods

This study was conducted retrospectively at a single center, in the emergency department of a tertiary university hospital. Our hospital is a tertiary center with a capacity of 1,200 beds where all internal and surgical specialty areas provide services 24 hours a day. The study was initiated after obtaining approval from the local ethics committee.

For the study, patients who underwent an in-hospital cardiac arrest between January 1, 2015, and December 1, 2020, after admission to the adult emergency department were examined. Patients in whom return of spontaneous circulation (ROSC) was achieved after CPR and thorax CT imaging were performed for diagnostic purposes were included in the study. These patients were divided into two groups: those who received manual resuscitation and those who received mechanical resuscitation. In our hospital, manual or mechanical CPR can be performed according to the physician’s decision. Manual CPR is performed only in patients who are not suitable for mechanical CPR (e.g., patients with morbid obesity, severe cachexia, and chest wall deformity) or when the mechanical device is unavailable or out of order. Manual CPR is also performed while the mechanical CPR device is prepared for use by trained personnel. Patients aged <18 years, with traumatic cardiac arrests, nonwitnessed cardiac arrests, and patients who could not be scanned by CT were excluded from the study. In addition, patients who underwent CT before the cardiac arrest were sent directly to the coronary angiography laboratory without performing CT because of ST-segment elevation myocardial infarction (STEMI), underwent one cycle of CPR, had a witnessed arrest, and rapidly defibrillated were excluded from the study.

Advanced cardiovascular life support training programs are organized at regular intervals in the emergency department accredited by the country’s accreditation agency [16]. Care was provided in all patients with cardiac arrest in accordance with the Utstein protocol [17]. In case of persistent ST-segment elevation in patients in whom ROSC was achieved after cardiac arrest, the patients were referred for angiography.

In line with the previous studies, CPR durations were determined to be <20 min and >20 min for the evaluation of the relationship between complications and CPR duration in our study [14].

Although manual CPR was performed in all patients who underwent CPR in the emergency department before 2018, the LUCAS 2 Chest Compression System (Physio-Control Inc./Jolife AB, Lund, Sweden) device was used after 2018.

Demographic data, vital parameters at admission, examination findings and electrocardiographic rhythms at the time of admission, CPR method, CPR duration, complications, emergency department outcomes, hospitalization periods, hospitalization outcomes, and modified Rankin Scale (mRS) scores were recorded. 

Statistical analysis

Categorical data were described as percentages (%). The Shapiro-Wilk test was used to investigate the normality of the distribution of data. Continuous variables were reported as medians and interquartile ranges (IQR) according to the distribution pattern of the variables. In the comparisons between groups that did not conform to the normal distribution, the Mann-Whitney U test was used for cases with two groups. Pearson’s chi-square and Fisher's exact chi-square analyses were used for analyzing the cross-tables created. Analyses were performed with the IBM Statistical Package for the Social Sciences (SPSS) 21.0 (IBM Corp., 2012, Armonk, NY) software. P <0.05 was considered statistically significant.

## Results

**Figure 1:**
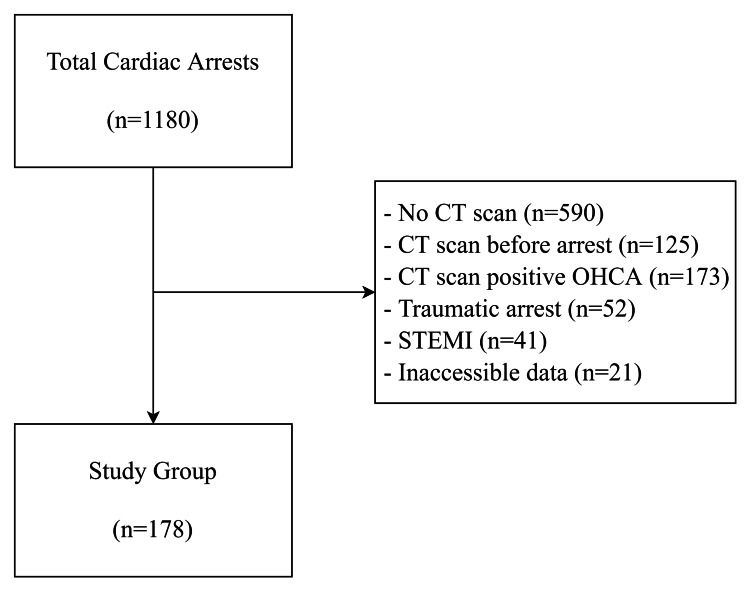
The number of arrest patients in the emergency department CT: computed tomography, OHCA: out-of-hospital cardiac arrest

The number of patients who received CPR in the emergency department was 1,180. Imaging could be performed with tomography in 528 of them. CT imaging of 125 patients was performed before the cardiac arrest, and these patients had no post-resuscitation tomography imaging. In the study, 173 and 52 patients were excluded because of experiencing out-of-hospital and traumatic cardiac arrests, respectively; 21 patients were also excluded from the study because of ambiguity regarding the CPR method used and method changes during CPR. In total, 178 patients whose data could be fully accessed were included in the study (Figure 1).

The median age of the patients was 72.50 (IQR 65-80). Of the patients, 79 (44.4%) were women. The initially detected cardiac arrest rhythms were asystole in 140 (78.7%), pulseless electrical activity in 23 (12.9%), ventricular fibrillation in 14 (6.2%), and ventricular tachycardia in four (2.2%) patients. The most common complaint was shortness of breath (n = 79 patients; 44.4%; Table 1). Although manual resuscitation was performed in 131 (73.6%) patients, mechanical resuscitation was performed in 47 (26.4%). Demographic data and patient characteristics are presented in Table 1. Vital signs could be measured in 95 patients before the in-hospital cardiac arrest, and the analysis of these data is presented in Table 1. 

**Table 1:** Demographic data and characteristics of the patients IQR: interquartile range, PEA: pulseless electrical activity, VF: ventricular fibrillation, VT: ventricular tachycardia, SBP: systolic blood pressure, DBP: diastolic blood pressure, SpO2: oxygen saturation

	Manual (n=131)	Mechanical (n=47)	p-value
Age, median [IQR]	73.0 (65.0 - 81.0)	71.0 (65.0 - 77.5)	0.368
Female, n(%)	65 (49.6%)	14 (29.8%)	0.029
Initial Complaint n(%)			
Dyspnea	56 (42.7%)	23 (48.9%)	0.774
Altered mental status	34 (26.0%)	10 (21.3%)	
Syncope	20 (15.3%)	4 (8.5%)	
Angina pectoris	16 (12.2%)	7 (14.9%)	
Back pain	3 (2.3%)	2 (4.3%)	
Abdominal pain	2 (1.5%)	1 (2.1%)	
Arrest rhythm n(%)			
Asystole	103 (78.6%)	37 (78.7%)	0.168
PEA	14 (10.7%)	9 (19.1%)	
VF	10 (7.6%)	1 (2.1%)	
VT	4 (3.1%)	0 (0%)	
Vital parameters	n=65	n=30	
SBP, median [IQR]	90.0 (70.0 - 120)	90.0 (80.0 – 118.0)	0.926
DBP, median [IQR]	60.0 (40.0 - 70.0)	50.0 (50.0 - 70.0)	0.685
Pulse, median [IQR]	104.0 (79.0 - 130)	118.0 (89.3 – 130.0)	0.294
SpO_2_, median [IQR]	78.0 (70.0 - 88.0)	75.0 (69.3 - 88.0)	0.860

Comparisons of CPR durations revealed no statistically significant difference between the mechanical and manual resuscitation methods (p = 0.313). There was no statistically significant in terms of lung contusion, rib fracture, and pneumothorax according to the CT scans of the patients after CPR (p = 0.914, p = 0.565, and p = 0.448, respectively). In the manual resuscitation group, one patient had a sternum fracture and another had hemothorax. There was no aortic dissection, solid organ injury, or cardiac injury detected in either group (Table 2). 

**Table 2:** CPR duration, CT findings, and the outcomes from the hospital according to the CPR method IQR: interquartile range, CPR: cardiopulmonary resuscitation, CT: computed tomography, ED: emergency department

	Manual (n=131)	Mechanical (n=47)	p
CPR duration, min, median [IQR]	10.0 [6.0-20.0]	14.0 [8.0-22.0]	0.313
CT Findings, n(%)			
Lung contusion	49 (37.4%)	18 (38.3%)	0.914
Rib fracture	14 (10.7%)	3 (6.4%)	0.565
Pneumothorax	8 (6.1%)	1 (2.1%)	0.448
Hemothorax	1 (0.8%)	0 (0%)	-
Sternum fracture	1 (0.8%)	0 (0%)	-
Exitus in ED, n(%)	16 (12.2%)	7 (14.9%)	0.638
Discharge from hospital, n(%)	8 (7.0%)	6 (15.0%)	0.196
Length of stay, hours, median [IQR]	72.0 [24.0-216.0]	48.0 [15.0-342.0]	0.954

In total, 16 (12.2%) patients in the manual resuscitation group and seven (14.9%) in the mechanical resuscitation group died in the emergency department (p = 0.638). Of the remaining 155 patients, eight (7.0%) from the manual resuscitation group and six (15.0%) from the mechanical resuscitation group were discharged from the hospital (p = 0.196). There was no significant difference in terms of the duration of hospital stay (Table 2).

In the manual resuscitation group, the mRS scores of the patients discharged were 2 in one patient, 3 in four patients, 4 in one patient, and 5 in two patients. In the mechanical group, the mRS scores were 3 in one patient, 4 in four patients, and 5 in one patient. 

**Figure 2:**
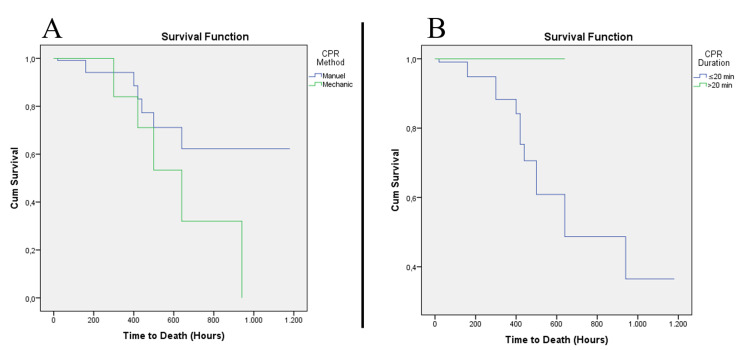
Evaluation chart based on the dates of death A) Life table of the patients according to CPR method; B) Life table of the patients according to CPR duration CPR: cardiopulmonary resuscitation

In the evaluation made according to the CPR duration, the number of patients who received CPR for ≤20 min was 126 (70.8%), whereas the number of those who received CPR >20 min was 52 (29.2%). Complications were more common in those patients who received CPR for >20 min (p<0.001) (Table 3). Nine (17.3%) of the patients who received CPR for >20 min and 14 (11.1%) of those who received CPR for <20 min died in the emergency department (p = 0.262). Of the 155 patients who were hospitalized, 14 who received CPR for <20 min were discharged, whereas no patient who received CPR for >20 min was discharged (p = 0.011). Similarly, a statistically significant difference was observed in the duration of hospital stay (p = 0.033). An evaluation chart made according to the dates of death is shown in Figure 2.

**Table 3:** CPR methods, CT findings, and the outcomes from the hospital according to CPR duration IQR: interquartile range, CPR: cardiopulmonary resuscitation, CT: computed tomography, ED: emergency department

	≤ 20 min (n=126)	> 20 min ( n=52)	p
Manual CPR, n(%)	93 (73.8%)	38 (73.1%)	0.920
Mechanical CPR, n(%)	33 (26.2%)	14 (26.9%)	
CT Findings, n(%)			
Lung contusion	30 (23.8%)	37 (71.2%)	<0.001
Rib fracture	7 (5.6%)	10 (19.2%)	0.009
Pneumothorax	4 (3.2%)	5 (9.6%)	0.125
Hemothorax	8 (6.1%)	1 (2.1%)	-
Sternum fracture	1 (0.8%)	0 (0%)	-
Exitus in ED, n(%)	14 (11.1%)	9 (17.3%)	0.262
Discharge from hospital, n(%)	14 (12.5%)	0 (0.0%)	0.011
Length of stay, hours, median [IQR]	24.0 [24.0-96.0]	96.0 [24.0-288.0]	0.033

## Discussion

In this study, according to our results, complications related to mechanical and manual CPR methods were at similar rates. In addition, prolonged CPR increased the mortality rate regardless of the method.

Complications resulting from CPR after a cardiac arrest are crucial and continue to be investigated because they affect the mortality and survival of these patients and have judicial and medical consequences for the attending physicians [15]. Whether mechanical compression devices, which have been used increasingly in recent years, increase these complications and survival rate is controversial [18-19].

The median age of the patients included in our study was 72.50, and 44.4% of these patients were women. In a study in which Petrovich et al. investigated complications associated with mechanical compression, the median age was 63 years, and 70% of patients were male [20]. We believe that these results differed from our results because of the sociodemographic characteristics of the region where the study was conducted.

In our study, the most common complaint in witnessed cardiac arrests was shortness of breath. In a study conducted by Iglesies et al., this complaint was STEMI [21]. Acute myocardial infarction was more common possibly because out-of-hospital cardiac arrests were evaluated in that study. However, the patients in our study included those who underwent a cardiac arrest in the emergency department of our hospital, thus the difference might have been because of the presence of other critical patient groups. As patients with STEMI were referred directly for angiography in our study, no additional intervention was performed in those patients.

In a study conducted by Smekal et al. in 2009, no significant difference was found between mechanical and manual compressions in terms of traumatic pathologies [19]. In a study by Koster et al., LUCAS did not cause serious and life-threatening injuries compared to manual compression [22]. Conversely, in a study by Iglesies et al., traumatic lesions were more common in the mechanical compression group [21]. In a retrospective cohort study by Koga et al., life-threatening injuries were more common in the mechanical compression group [23]. In those studies, it was considered that ROSC took longer in patients who received mechanical CPR, and therefore, complications were more common in the mechanical group. Similarly, detection of a higher number of fatal complications was possibly because of the larger number of patients included.

In the study conducted by Smekal et al. in 2014, rib fractures were more common in patients who received mechanical compression [24]. In a study by Wik et al., injuries such as rib fracture and pneumothorax were more common in the mechanical compression group [25]. In our study, no significant difference was found between the patients in the mechanical and manual resuscitation groups in terms of complications. This might be because most of the studies were conducted in autopsy studies and the complications were more common in patients who died.

In a meta-analysis performed by Bonnes et al., survival rates until hospitalization were higher in patients who received mechanical compression, but no significant difference was found in terms of survival and neurological outcomes at discharge [13]. In our study, there was no difference between the mechanical and manual resuscitation groups in terms of survival until hospitalization, survival at discharge, and neurological outcomes of the patients as assessed by the mRS. Although there was no statistically significant difference, when we considered the rate of discharge from hospital, we observed that this rate was higher in patients who received mechanical compression. Similarly, in circulation improving resuscitation care and LUCAS in cardiac arrest studies, there was no difference between mechanical and manual compressions in terms of survival and discharge with good neurological functions [25-27]. Manual and mechanical resuscitation resulted in similar outcomes in our study, which was consistent with the findings of these large-scale studies.

In a study by Kaldirım et al., complications such as rib fracture, pneumothorax, contusion, and cardiac laceration increased with increased CPR duration [28]. Similarly, in our study, a significant increase was found in rib fracture and lung contusion rates when the duration of CPR exceeded 20 min. However, we found that this had no effect on survival in the emergency department. In a study by Reynolds et al., the rate of survival from hospitalization to discharge and good neurological outcomes at discharge decreased rapidly with every minute of CPR [29]. In our study, patients could not be discharged when CPR duration exceeded 20 min. The prolongation of CPR duration does not affect survival, but it affects the efforts of the rescuers and remains an important ethical issue.

Limitations

The primary limitation of our study lies in its single-center and retrospective design. As the use of mechanical compression devices was initiated in 2018, cases before 2018 could not be included in the study. CT was not performed in patients in whom spontaneous circulation returned after the in-hospital cardiac arrest, and thus these cases were not included in our study. In addition, patients in whom spontaneous circulation did not return were excluded from the study. The confounding additional pathologies of patients, such as underlying lung parenchymal disease and osteoporosis before the cardiac arrest was not known, which might have also limited the analysis. As the device we use in our center is LUCAS, no information could be obtained about the possible complications of other mechanical compression devices.

## Conclusions

In this study, no significant difference was observed in terms of increased complications in patients who received mechanical compression as compared with those who received manual compression. The rate of hospital discharge was higher in patients who received mechanical compression but there was no statistically significant difference. According to our results, mechanical compression devices should be used more frequently. Further prospective and multicenter studies are warranted to identify and prevent complications that can be detected by CT after manual and mechanical CPR and to evaluate their effects on survival.
